# Yijin-Tang Attenuates Cigarette Smoke and Lipopolysaccharide-Induced Chronic Obstructive Pulmonary Disease in Mice

**DOI:** 10.1155/2022/7902920

**Published:** 2022-01-05

**Authors:** Jinhyung Rho, Chang-Seob Seo, Eun-Ju Hong, Eun Bok Baek, Eunhye Jung, Suyoung Park, Mee-Young Lee, Hyo-Jung Kwun

**Affiliations:** ^1^Department of Veterinary Pathology, College of Veterinary Medicine, Chungnam National University, Daejeon, Republic of Korea; ^2^KM Science Research Division, Korea Institute of Oriental Medicine, Daejeon, Republic of Korea

## Abstract

**Background:**

Chronic obstructive pulmonary disease (COPD) refers to a lung disorder associated with symptoms of dyspnea, cough, and sputum production. Traditionally, Yijin-tang (YJT), a mixture of *Pinellia ternate*, *Poria cocos*, ginger, Chinese liquorice, and tangerine peel, has been prescribed for the treatment of respiratory system diseases caused by dampness phlegm. This experiment investigated the therapeutic effect of YJT in a mouse model of cigarette smoke (CS)- and lipopolysaccharide (LPS)-induced COPD.

**Methods:**

COPD was induced by exposing mice to CS for 1 hour per day for 8 weeks, with intranasal delivery of LPS on weeks 1, 3, 5, and 7. YJT was administered at doses of 100 and 200 mg/kg 1 hour before CS exposure for the last 4 weeks.

**Results:**

YJT significantly suppressed CS- and LPS-induced increases in inflammatory cell counts and reduced interleukin-1 beta (IL-1*β*), IL-6, tumor necrosis factor-alpha (TNF-*α*), and monocyte chemoattractant protein-1 (MCP-1) levels in bronchoalveolar lavage fluid (BALF) and lung tissue. In addition, YJT not only decreased airway wall thickness, average alveolar intercept, and lung fibrosis, but it also suppressed the expression of matrix metallopeptidase (MMP)-7, MMP-9, and transforming growth factor-B (TGF-*β*) and collagen deposition. Moreover, YJT suppressed phosphorylation of nuclear factor-kappa B (NF-*κ*B) as well as expression of cyclooxygenase-2 (COX-2) and inducible nitric oxide synthase (iNOS).

**Conclusion:**

Collectively, our findings show that YJT attenuates respiratory inflammation and airway remodeling caused by CS and LPS exposure; therefore, therapeutic applications in COPD can be considered.

## 1. Background

Chronic obstructive pulmonary disease (COPD) refers to a lung disorder characterized by disturbed airflow accompanied by dyspnea, cough, and sputum production [1, 2]. Persistent airflow resistance is caused by a combination of bronchiolar inflammatory disease, structural destruction of the parenchyma, and emphysema [2]. The chronic airway inflammation associated with COPD is characterized by the extensive migration of various subsets of leukocytes, including neutrophils, eosinophils, macrophages, and lymphocytes. Inhalation of air pollutants triggers the engagement of pattern-recognition receptors, causing the recruitment of leukocytes by releasing numerous chemokines and proinflammatory cytokines [3]. These secreted compounds mediate to promote the chronic, progressive airflow limitations that accompany fibrosis and inflammation.

The occurrence of COPD has consistently increased proportionally with increases in the smoking population. Recent available treatments, such as corticosteroids, *β*2-agonists, and phosphodiesterase (PDE) type 4 inhibitors, are highly effective to clinical signs, including dyspnea and exercise intolerance, and mitigate the more extreme manifestations of the disease [4, 5]. However, such therapies cannot inhibit disease progression and carry the potential for drug intolerance and severe side effects [5, 6]. Therefore, alternative agents with higher efficacy and lower toxicity for the treatment of COPD are being explored.

Yijin-tang (YJT; *Erchen-tang* in Chinese and *Nichin-to* in Japanese), a traditional prescription composed of *Pinellia ternate, Poria cocos*, ginger, Chinese liquorice, and tangerine peel [7, 8] has been used for the prevention and treatment of diverse symptoms. The major bioactive components of YJT include flavonoids (such as liquiritin, liquiritigenin, hesperidin, rutin, naringin, neohesperidin, and poncirin), triterpenoids (such as glycyrrhizin, pachymic acid, and eburicoic acid), phenolic acids (such as homogentisic acid), and pungent principles (such as 6-gingerol and 6-shogaol) [9]. These compounds, originating from the individual herbal medicine constituents, possess various pharmacological properties, including anticancer, anti-inflammatory, antibacterial, and antioxidant activities [10–16]. Accordingly, YJT has been widely used to treat symptoms including nausea, vomiting, gastroduodenal ulcers, and chronic gastritis [10]. In oriental medicine, dampness phlegm refers to the blend of phlegm and internal dampness as an entity that causes disease, in which internal dampness is characterized by impeded flow and turbidity of body fluid due to increased viscosity resulting in feelings of heaviness. Phlegm is a pathological over secretion of body fluid in the respiratory tract that has been linked to various diseases, including COPD. However, no scientific experiments have been conducted to investigate the therapeutic effects of YJT on COPD. In our study, we explored whether YJT has protective effects against COPD by using a mouse model of CS- and LPS-induced COPD.

## 2. Methods

### 2.1. Plant Materials

The five raw plant materials that constitute YJT, listed in Table S1, were gained from the herbal markets: HMAX Co. Ltd. (Jecheon, Korea) and Omniherb Co. Ltd. (Daegu, Korea). Each herbal medicine was validated by Dr. Goya Choi, the Herbal Medicine Resources Research Center, Korea Institute of Oriental Medicine (KIOM; Naju, Korea), based on “The Dispensatory on the Visual and Organoleptic Examination of Herbal Medicine” [17]. The voucher specimen (2008KE08–1 to 2008KE08–5) has been deposited at the Herbal Medicine Research Division, KIOM.

### 2.2. Chemicals and Reagents

The following six standard compounds used for quantitative analyses were purchased from the indicated suppliers: liquiritin apioside (Cat No. 74639-14-8, purity: 98.0%) from Shanghai Sunny Biotech (Shanghai, China); glycyrrhizin (Cat No. 1405-86-3, purity: 99.4%), liquiritin (Cat No. 551-15-5, purity: 99.6%), and 6-gingerol (Cat No. 23513-08-8, purity: 98.3%) from Wako Chemicals (Osaka, Japan); and narirutin (Cat No. 14259-46-2, purity: 99.5%) and hesperidin (Cat No. 520-26-3, purity: 98.6%) from Biopurify Phytochemicals (Chengdu, China). The chemical structures of these reference standard compounds, used for the quantitative high-performance liquid chromatography (HPLC) analysis of YJT samples, are shown in Supplementary Figure 1. Solvents (methanol, acetonitrile, and water) used for simultaneous analyses were HPLC grade and were purchased from JT Baker (Phillipsburg, NJ, USA). Formic acid (ACS reagent grade, Cat No. 64-18-6, and purity: 100.0%) and dimethyl sulfoxide (ACS reagent grade, Cat No. 67-68-5, and purity: 99.9%) were purchased from Merck KGaA (Darmstadt, Germany).

### 2.3. Preparation of YJT Water Extract

An aqueous extract of YJT was prepared by first mixing the five herbs—*Pinellia* tuber (1272.74 g), Citri Unshius (636.36 g), Pericarpium (636.36 g), Glycyrrhizae Radix et Rhizoma (318.18 g), and Zingiberis Rhizoma Recens (636.36 g)—and then extracting the mixture with 3.5 L of distilled water. Extraction and pretreatment procedures were performed according to a previously reported method [18]. The extract was freeze-dried, and a total of 640.3 g of powder (18.3% yield) was obtained as a final product.

### 2.4. Preparations of Sample and Standard Solutions for HPLC Analysis

Sample solutions (10 mL) for quantification of herbal components were prepared at concentrations of 1 mg/mL (liquiritin apioside, liquiritin, narirutin, hesperidin, and glycyrrhizin) or 10 mg/mL (6-gingerol) in 70% methanol. Sample solutions thus prepared were filtered using a 0.2 *μ*m membrane (Pall Life Sciences, Ann Arbor, MI, USA) and analyzed by HPLC.

The standard solution for each analyte was prepared at a level of 1,000 *μ*g/mL in methanol (liquiritin apioside, liquiritin, narirutin, glycyrrhizin, and 6-gingerol) or methanol-DMSO (1 : 1) (hesperidin), and then stored at ∼4°C until use.

### 2.5. HPLC System and Conditions for Simultaneous Quantitative Analysis of Six Marker Analytes

The six marker analytes were quantitatively analyzed as described previously (Seo and Shin, 2017) using a Shimadzu Prominence LC-20A series (Kyoto, Japan) combined with a photodiode array detector. A reverse-phase SunFire C18 column (Waters, Milford, MA, USA) was used, and samples were gradient eluted with mobile phases consisting of distilled water and acetonitrile, both containing 0.1% (v/v) formic acid. Other specific analysis conditions are shown in Table S2.

### 2.6. Animals

Male C57BL/6 mice (7 weeks of age) were bought from Orient Bio (Seongnam, Korea). During the experiment, the mice were raised in constant conditions such as a temperature of 22°C ± 2°C, a relative humidity of 50% ± 5%, and a 12 h light/dark cycle. Standard rodent chow and filtered water were provided freely. The mice were quarantined and acclimatized for one week prior to the experiments. Experiments that involved animals in this study were reviewed and approved by the Animal Ethics Committee (Daejeon, South Korea).

### 2.7. Induction of COPD

The animals were classified into five groups after the acclimatization period (*n* = 7-8 per group). The five groups are as follows: (i) normal control (CON) group; (ii) CS exposure/LPS intranasal administration (CS + LPS) group; (iii) CS exposure/LPS administration + 5 mg/kg roflumilast p.o (RO) group (used as a positive control group); (iv) CS exposure/LPS administration + 100 mg/kg YJT p.o (YJT100) group; and (v) CS exposure/LPS administration + 200 mg/kg YJT p.o (YJT200) group. Cigarette smoke (CS) was generated by burning 3R4F research cigarettes manufactured from the Tobacco and Health Research Institute at the University of Kentucky (KY, USA). We exposed mice to CS from 8 cigarettes for 60 min/day in an exposure chamber with air pumps providing fresh, clean air. The CS lasted for 8 weeks with 5 days in a week. LPS (10µg per mouse) was anesthetized and exposed to animals via intranasal administration a total of four times on weeks 1, 3, 5, and 7. Roflumilast and YJT were administered to animals via p.o 1 hour before CS exposure for the last 4 weeks.

### 2.8. Collection of Blood and Bronchoalveolar Lavage Fluid (BALF)

The animals were euthanized 48 hours after the last induction of CS. Animals were fasted for 12 hours before the sacrifice. Serum was collected via the caudal vena cava and centrifuged at 1500 rpm for 15 min. It was stored at −80°C deep freezer for further investigations. BALF was collected through tracheal intubation with the help of a cannula. Bronchoalveolar was washed by 1.5 mL of Dulbecco's phosphate-buffered saline (DPBS), pumping three times. It was then centrifuged, with its supernatant collected and kept in a 70°C deep freezer. Cell pellets were stained trypan blue and evaluated by counting alive cells in five squares of a hemocytometer. In addition, Diff-Quik stain (B4132-1A; IMEB Inc., IL, USA) was performed with another aliquot of BALF (100 *μ*L) on a slide. The cells were centrifuged by the Cytospin system (Hanil Science Industrial, Seoul, Korea), followed by drying slides. Following fixation, cells were stained with Diff-Quik stain reagents in accordance with the instructions.

### 2.9. Histopathological Examination

Lung tissues were fixed in 10% neutral buffered formalin (NBF) for 24 hours. The tissues were treated as a part of the routine tissue process and stained with hematoxylin and eosin. In order to investigate pulmonary change, mean linear intercept (MLI) was measured in five pictures of randomly selected 4 animals in each group according to the previous study [19]. 5 equally-spaced horizontal lines in 20X magnification were viewed. Then, the total length of all lines counted was divided by the total number of intercepts per field. The mean thickness of the basement membrane of the bronchiole was measured at 20X of a randomly selected similar-sized bronchiole. The sirius-red stain was conducted by incubating for 50 minutes at room temperature followed by rehydration.

### 2.10. Immunohistochemistry (IHC) Assay

To perform IHC, primary antibody against matrix metallopeptidase (MMP)-7 (Abcam, Cambridge, UK) was applied overnight in a 200 : 1 diluted solution according to the manufacturer's guide. The positive area was calculated in each lung tissue of at least 4 animals per group.

### 2.11. Enzyme-Linked Immunosorbent Assay (ELISA)

The BALF level of interleukin (IL)-6 and tumor necrosis factor (TNF)-*α* were investigated by commercially available kits (R&D Systems, Minneapolis, MN, USA). Following incubation for 10 minutes in the dark, the level was determined by optical absorbance at 450 nm with a microplate reader (Infinite m200pro; Tecan, Männedorf, Switzerland).

### 2.12. RNA Extraction and Real-Time PCR Analysis of mRNA Expression

Total RNA of the lungs was isolated by TRIzol reagent (Invitrogen, Carlsbad, CA, USA) after homogenization of lung tissue. The RNA level was determined by detecting optical intensity at a 260 nm wavelength, and its purity was detected by the ratio of 260 nm to 280 nm absorbance. cDNA was synthesized from 1 *μ*g of total RNA with a reverse transcription kit (Toyobo, Japan) according to instructions. A polymerase chain reaction (PCR) was performed on an Applied Biosystems 7500 Real-Time PCR System (Life Technologies, CA, USA) using SYBR Green PCR Master Mix (Applied Biosystems, CA, USA) according to the instructions. The PCR primers utilized in these experiments are listed in Table 1. PCR data were analyzed using Applied Biosystems 7500 Real-Time PCR System software (Applied Biosystems), and the ratio of the fold change in expression of the target gene to the endogenous control (GAPDH) was calculated using the 2^−ΔΔCt^ method, as described previously (Park et al., 2018). [20].

### 2.13. Western Blot Analysis

The lung samples were grounded in a RIPA lysis buffer (Cell Signaling Technology, MA, USA) and combined with inhibitor cocktails. We measured equivalent amounts of lung proteins (30 *μ*g), loaded by 8% SDS-PAGE, electrophoresed in 60V, and transferred the proteins into polyvinylidene fluoride (PVDF) at 250V for 2 hours. The transferred membranes were blocked with phosphate-buffered saline (PBS) containing 0.05% Tween 20 (PBST) and 5% skim milk for 1 hour and were incubated overnight at 4°C with antinuclear factor-kappa B (NF-*κ*B), antiphospho-NF-*κ*B (Cell Signaling Technology, MA, USA), anticyclooxygenase-2 (COX-2; Abcam, Cambridge, UK), inducible nitric oxide synthase (iNOS; Abcam), or anti-*β*-actin (Sigma-Aldrich, MO, USA). We incubated membranes with antibodies for 1 hour and detected protein expression by luminograph (ATTO, Tokyo, Japan).

### 2.14. Statistical Analysis

Data are expressed as means ± standard deviation (SD). Statistical comparisons were made by one-way analysis of variance (ANOVA) using GraphPad Prism 6 (GraphPad, CA, USA). If the data is significant, a post hoc test with Tukey adjustment was performed. Statistical significance was determined by *P* value <0.05.

## 3. Results

### 3.1. Optimization of HPLC Chromatographic Conditions and Quantitative Analysis of Six Marker Analytes

**Figure 1:**
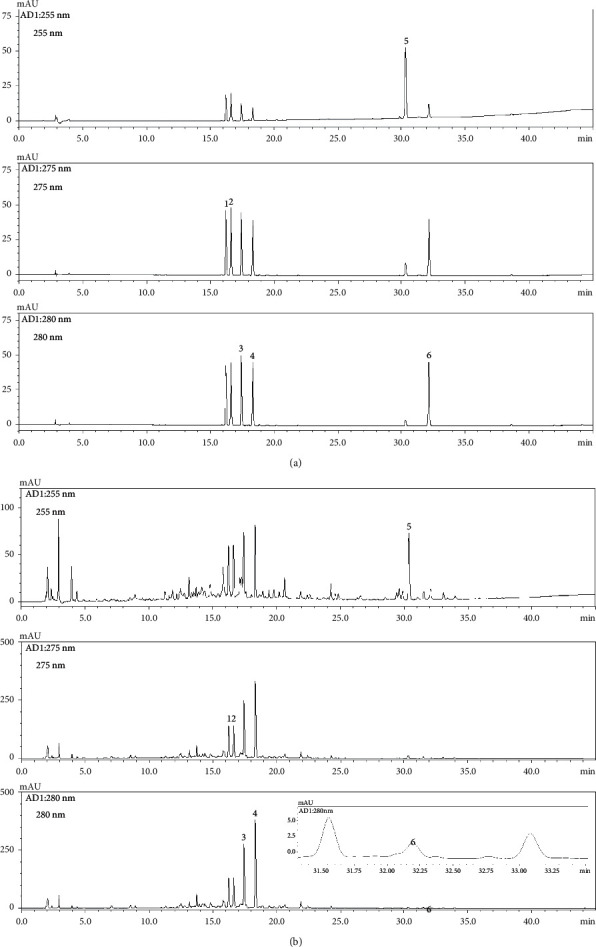
An HPLC chromatogram of standard solution (a) and Yijin-tang sample (b). Liquiritin apioside (1), liquiritin (2), narirutin (3), hesperidin (4), glycyrrhizin (5), and 6-gingerol (6).

Optimal analysis conditions for the six marker analytes (liquiritin apioside, liquiritin, narirutin, hesperidin, glycyrrhizin, and 6-gingerol) in the YJT sample were established as follows: column, SunFire C_18_ (250 mm × 4.6 mm, 5 *μ*m); column oven temperature, 40°C; mobile phase system, distilled water-acetonitrile (both containing 0.1% formic acid). Applying these analysis conditions to the YJT sample resulted in the separation of all components within 35 minutes (Figure 1). The system suitability factors, capacity factor (*k′*), selectivity factor (*α*), resolution (*Rs*), number of theoretical plates (*N*), and tailing factor (*Tf*), of the assay were also acceptable (Table S3).

A calibration curve for quantitative analysis of the six marker components was prepared as the ratio of peak area (*y*) versus the corresponding concentration (*x*) over the range shown in Table 2. The resulting coefficient of determination was ≥0.9999, showing excellent linearity. The limit of detection (LOD) and limit of quantification (LOQ) ranges were calculated to be 0.03–0.13 *μ*g/mL and 0.09–0.40 *μ*g/mL, respectively. These results, summarized in Table 2, demonstrate that the established HPLC analytical method is suitable for quantitative determination of the six marker analytes in the YJT sample.

Each of the six analytes in YJT samples was quantified by reference to the corresponding calibration curve using the indicated HPLC analysis conditions, with glycyrrhizin quantified at 255 nm, liquiritin apioside and liquiritin at 275 nm, and narirutin, hesperidin, and 6-gingerol at 280 nm. The concentrations of the six marker analytes in YJT samples ranged from 0.28 to 12.88 mg/g (Table 3).

### 3.2. Effects of YJT on Pulmonary Histological Injury

**Figure 2:**
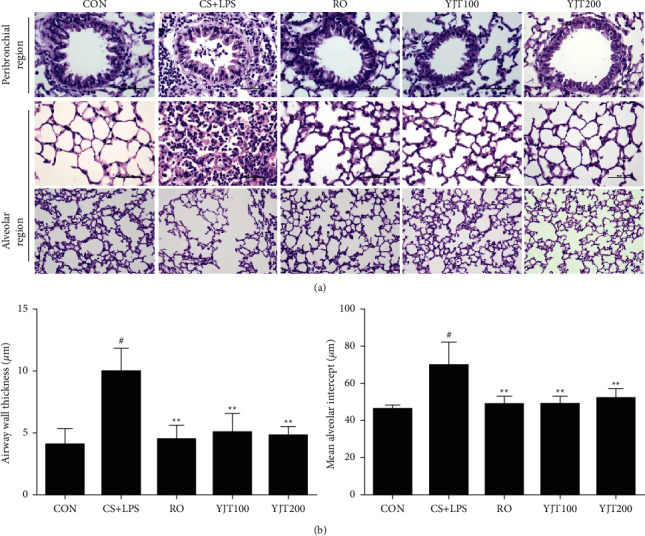
YJT inhibited CS- and LPS-induced pulmonary damage. Histological view of the lung and analysis. (a) H&E staining. (b) Airway wall thickness. (c) Average alveolar intercept, representing the degree of emphysema, quantified according to the mean linear intercept (MLI). CON, control; CS + LPS, CS/LPS-exposed mice; RO, roflumilast (10 mg/kg) + CS/LPS-exposed mice; YJT100, YJT (100 mg/kg) + CS/LPS-exposed mice; YJT200, YJT (200 mg/kg) + CS/LPS-exposed mice. Results are presented as means ± SD (^#^*P* < 0.05, ^##^*P* < 0.01 compared with the CON group; ^*∗*^*P* < 0.05, ^*∗∗*^*P* < 0.01 compared with the CS + LPS group).

To evaluate whether YJT affects histopathological changes induced by exposure to CS and LPS, we stained the alveolar region and peribronchial region in lung tissue with H and E. As shown in Figure 2, CS- and LPS-exposed animals showed severe infiltration of immune cells in peribronchiolar and alveoli and increased airway wall thickness and average alveolar intercepts, reflecting the degree of emphysema. However, inflammatory cell infiltration, airway wall thickness, and average alveolar intercept declined in the roflumilast- and YJT-treated groups than those in the CS- and LPS-exposed groups (Figure 2).

### 3.3. Effects of YJT on Inflammatory Cells in BALF

**Figure 3:**
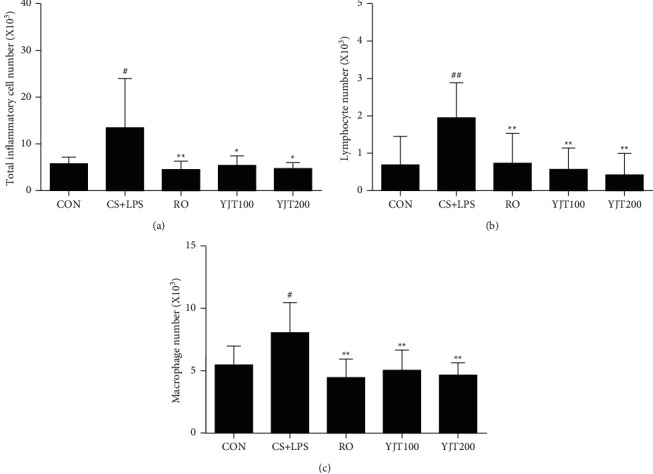
YJT decreased the number of inflammatory cells in BALF. (a) Number of total cells. (b) Number of lymphocytes. (c) Number of macrophages. CON, control; CS + LPS, CS/LPS-exposed mice; RO, roflumilast (10 mg/kg) + CS/LPS-exposed mice; YJT100, YJT (100 mg/kg) + CS/LPS-exposed mice; YJT200, YJT (200 mg/kg) + CS/LPS-exposed mice. Results are presented as means ± SD (^#^*P* < 0.05, ^##^*P* < 0.01 compared with the CON group; ^*∗*^*P* < 0.05, ^*∗∗*^*P* < 0.01 compared with the CS + LPS group).

To investigate the effects of YJT on inflammatory responses triggered by CS and LPS, we determined cell numbers and subclass status in BALF. The number of total cells, lymphocytes, and macrophages in BALF were determined. As a result, the number of total cells, lymphocytes, and macrophages increased in BALF from CS- and LPS-exposed mice compared to that in control mice (Figure 3). Notably, the number of total cells, lymphocytes, and macrophages in BALF was meaningfully decreased in animals treated with roflumilast or YJT compared to CS- and LPS-exposed animals (Figure 3).

### 3.4. Effects of YJT on IL-6 and TNF-*α* Levels in BALF

**Figure 4:**
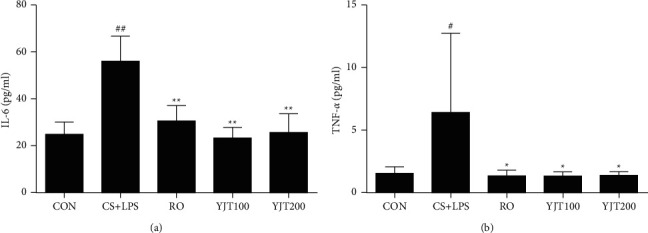
YJT reduced proinflammatory cytokines in BALF. (a) Concentration of IL-6. (b) Concentration of TNF-*α*. CON, control; CS + LPS, CS/LPS-exposed mice; RO, roflumilast (10 mg/kg) + CS/LPS-exposed mice; YJT100, YJT (100 mg/kg) + CS/LPS-exposed mice; YJT200, YJT (200 mg/kg) + CS/LPS-exposed mice. Results are presented as means ± SD (^#^*P* < 0.05, ^##^*P* < 0.01 compared with the CON group; ^*∗*^*P* < 0.05, ^*∗∗*^*P* < 0.01 compared with the CS + LPS group).

As demonstrated in Figure 4, IL-6 and TNF-*α* levels increased markedly in the BALF of CS- and LPS-exposed group than that in control mice. This effect was attenuated by roflumilast- and YJT, which significantly decreased IL-6 and TNF-*α* levels in BALF compared with those in CS- and LPS-exposed mice (Figure 4).

### 3.5. Effects of YJT on mRNA Levels of L-1*β*, IL-6, TNF-*α*, and MCP-1 in Lung Tissues

**Figure 5:**
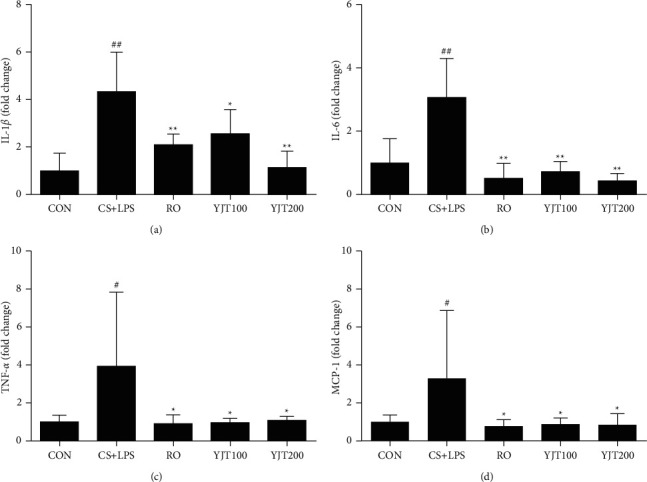
YJT downregulated mRNA expression of inflammatory cytokines in the lung. Levels of IL-1*β* (a), IL-6 (b), TNF-*α* (c), and MCP-1 (d), measured by real-time PCR. CON, control; CS + LPS, CS/LPS-exposed mice; RO, roflumilast (10 mg/kg) + CS/LPS-exposed mice; YJT100, YJT (100 mg/kg) + CS/LPS-exposed mice; YJT200, YJT (200 mg/kg) + CS/LPS-exposed mice. Results are presented as means ± SD (^#^*P* < 0.05, ^##^*P* < 0.01 compared with the CON group; ^*∗*^*P* < 0.05, ^*∗∗*^*P* < 0.01 compared with the CS + LPS group).

The levels of mRNA for IL-1*β*, IL-6, TNF-*α*, and MCP-1 notably increased in CS- and LPS-exposed mice compared with those in control mice (Figure 5). Consistent with their effects in BALF, roflumilast and YJT significantly decreased mRNA levels of IL-1*β*, IL-6, TNF-*α*, and MCP-1 in lung tissues than those in CS- and LPS-exposed mice (Figure 5).

### 3.6. Effects of YJT on NF-*κ*B Activation and Expression of COX-2 and iNOS

**Figure 6:**
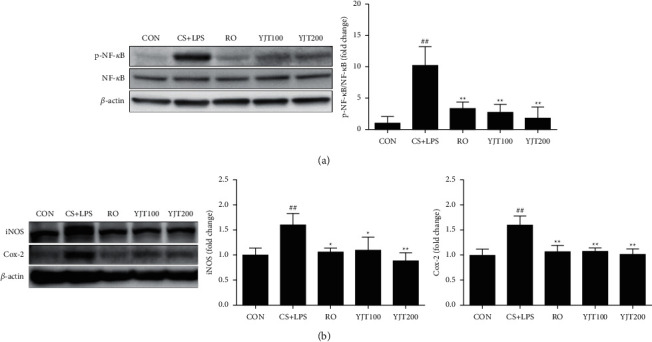
YJT decreased NF-*κ*B activity and expression of iNOS and COX-2. Phosphorylation of NF-*κ*B p65 (a) and expression of iNOS and COX-2 (b) in lung tissues, evaluated by western blot analysis. CON, control; CS + LPS, CS/LPS-exposed mice; RO, roflumilast (10 mg/kg) + CS/LPS-exposed mice; YJT100, YJT (100 mg/kg) + CS/LPS-exposed mice; YJT200, YJT (200 mg/kg) + CS/LPS-exposed mice. Results are presented as means ± SD (^#^*P* < 0.05, ^##^*P* < 0.01 compared with the CON group; ^*∗*^*P* < 0.05, ^*∗∗*^*P* < 0.01 compared with the CS + LPS group).

Phosphorylation of NF-*κ*B, COX-2, and iNOS is essential in inflammatory responses [21, 22]. As shown in Figure 6(a), phosphorylation of NF-*κ*B p65 in lung tissues was markedly increased in CS- and LPS-induced mice, an effect that was significantly reduced in the roflumilast and YJT treatment groups. Similarly, expression of COX-2 and iNOS were higher in CS- and LPS-exposed mice than that in control mice but attenuated in roflumilast or YJT-administered animals (Figure 6(b)).

### 3.7. Effects of YJT on Tissue Remodeling in Lung

**Figure 7:**
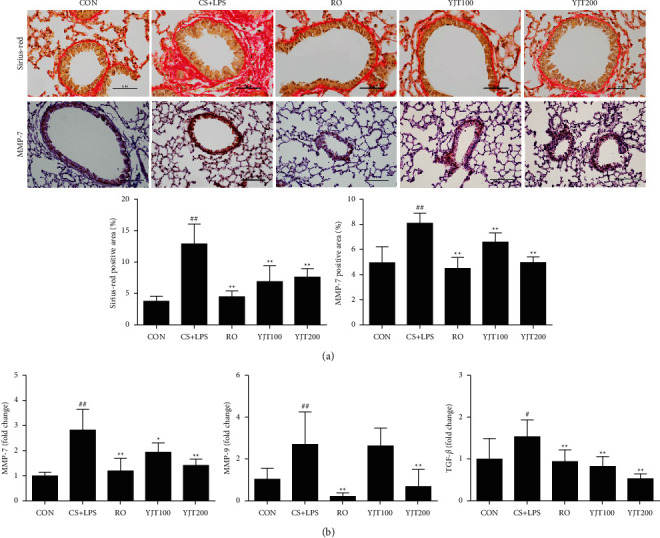
YJT suppressed the levels of MMPs and TGF-*β*. (a) Sirius-red staining and immunohistochemistry of MMP-7 (X200). (b) Expression of MMP-7, MMP-9, and TGF-*β* mRNA in the lung. CON, control; CS + LPS, CS/LPS-exposed mice; RO, roflumilast (10 mg/kg) + CS/LPS-exposed mice; YJT100, YJT (100 mg/kg) + CS/LPS-exposed mice; YJT200, YJT (200 mg/kg) + CS/LPS-exposed mice. Results are presented as means ± SD (^#^*P* < 0.05, ^##^*P* < 0.01 compared with the CON group; ^*∗*^*P* < 0.05, ^*∗∗*^*P* < 0.01 compared with the CS + LPS group).

Tissue remodeling in the damaged lung parenchyma is a critical mechanism of COPD [23]. The major architectural changes in the COPD lungs are increased deposition of interstitial extracellular matrix (ECM) and peribronchiolar fibrosis. Sirius-red staining showed increased peribronchiolar fibrosis in the lungs of CS- and LPS-exposed mice, changes that were markedly decreased in mice treated with roflumilast or YJT (Figure 7(a)). In addition, the positive area of MMP-7 increased in CS- and LPS-exposed mice, but this elevation was attenuated in roflumilast or YJT-treated animals (Figure 7(a)). Structural remodeling markers such as MMP-7, MMP-9, and TGF-*β* levels in the lungs of CS- and LPS-exposed animals were higher than those in control mice. Again, expression of these factors was markedly lower in animals treated with roflumilast or YJT compared with that of CS- and LPS-exposed mice (Figure 7(b)).

## 4. Discussion

In the present study, the protective effects of YJT on the progression of COPD were investigated via a mouse model of CS- and LPS-induced COPD. YJT significantly inhibited the lung tissue from recruiting inflammatory cells and producing cytokines in CS- and LPS-exposed BALF and lung tissues. It also attenuated airway wall thickening, emphysema and fibrosis, and effectively repressed the expression of MMPs and TGF-*β*, and the deposition of collagen in CS- and LPS-exposed mice.

COPD may be triggered by various immunologic responses in the lungs owing to chronic inhalation of irritants. CS contains numerous toxic chemicals, some of which may directly damage cell membranes [24], while others may indirectly damage pneumocytes by producing reactive oxygen species (ROS) [25–27]. Acting through either pathway, CS induces an inflammatory response that may secondarily cause emphysema or narrowing of small airways [5]. This chronic inflammation is characterized by the recruitment of inflammatory cells into the lungs, including neutrophils, macrophages, and lymphocytes. In turn, these cells produce proinflammatory cytokines and chemokines including TNF-*α*, IL-6, IL-1*β*, and MCP-1 [3]. TNF-*α*, mainly produced by macrophages, can promote the expression of CXCL10, a potent chemoattractant recruiting neutrophils, monocytes, and T-helper type 1 cells, consequently, causing pulmonary emphysema [28, 29]. IL-6, also predominantly secreted from macrophages, has neutrophil chemotactic effects, which may affect the severity of disease and prognosis in COPD patients [29, 30]. IL-1*β* promotes fibrosis in airway walls, enhances mucin production, and recruits lymphocyte aggregates in the airways [31]. MCP-1 is an important factor that recruits inflammatory cells to the lungs of COPD patients [32]. Our experiment showed that YJT treatment significantly attenuated the number of inflammatory cells and levels of proinflammatory in CS- and LPS-induced BALF and lung tissues of mice. These results show that YJT suppresses the inflammatory response associated with COPD.

The major histopathological features shown in biopsy specimens of COPD patients are characterized by abnormalities in the architecture of the lung including increased basement membrane, epithelial fibrosis, and increased thickness of the interstitium of the alveoli, with severe recruitment of inflammatory cells [33, 34]. These cellular events are called tissue remodeling. It involves severe modification of the ECM, which is critical for the physiological function of the lung [35]. MMPs, a family of endopeptidases, can degrade ECM proteins. Under normal state, MMPs play a role in the remodeling of the ECM and regulate immune responses by cleaving the inactivated cytokines and chemokines into active forms [35]. In COPD conditions, however, MMPs lead to excessive lung inflammation and abnormal tissue destruction [36]. In patients with COPD, the levels of MMP-7 and MMP-9 are increased in serum, plasma, sputum, BALF, and lung tissue, respectively [37–40]. These MMPs can destroy alveolar architecture, exacerbate inflammatory responses in lung tissues, and induce emphysema, causing loss of pulmonary function [41]. MMP-9 is also responsible for the activation and production of TGF-*β* and in alveolar epithelial cells [42]. TGF-4*β* is one of the most important growth factors involved in the pathogenesis of COPD. Activation of the TGF-*β* signaling pathway causes airway fibrosis and the development of emphysema, mainly by affecting the production and degradation of collagen in the ECM [43, 44]. In this study, YJT treatment attenuated collagen accumulation in bronchioles and reduced the thickening of alveolar walls and alveolar intercept. It also significantly suppressed elevated levels of MMP-7, MMP-9, and TGF-*β* in mice exposed to LPS and CS. These results suggest that the YJT may ameliorate COPD by suppressing airway remodeling.

In the present study, CS- and LPS-exposed mice exhibited enhanced phosphorylation of NF-*κ*B in comparison with control mice, and YJT treatment supressed activation of NF-*κ*B caused by CS and LPS exposure. TNF-*α*, IL-6 and IL-1*β* appear to amplify inflammation in COPD, in part through the activation of the transcription factor, NF-kB and MAPK, thereby leading to the increased expression of multiple inflammatory genes [45, 46]. It was previously shown that phosphorylation of NF-*κ*B is significantly increased in COPD patients and animal models [47]. Activation of NF-*κ*B is promoted by proinflammatory stimuli such as CS, LPS, and TNF-*α*, which cause degradation of the NF-*κ*B inhibitory protein, I*κ*B [22]. NF-*κ*B is then translocated to the nucleus, where it induces transcription of iNOS, COX-2, and MMP-9, all of which are key regulators of airway inflammatory responses [22]. Our results show that activation of NF-*κ*B is higher in CS- and LPS-exposed mice in association with elevated levels of MMP-9, iNOS, and COX-2 in lung tissue compared with controls. However, YJT-treated mice exhibited a marked reduction in phosphorylated NF-*κ*B, with decreases in MMP-9, iNOS, and COX-2 relative to CS- and LPS-exposed mice. These results suggest that YJT is capable of suppressing inflammatory responses and airway remodeling caused by CS and LPS exposure, in part by downregulating NF-*κ*B activation.

## 5. Conclusion

In short, our study demonstrated that YJT provides a protective effect against LPS- and CS-induced COPD in a mouse model by reducing inflammatory responses and airway remodeling, effects that may be related to suppression of the NF-*κ*B pathway. Therefore, our study demonstrates that YJT may be a potential agent for the treatment of COPD.

## Figures and Tables

**Table 1:** Primer sequences.

Target	Sequence
IL-1*β*	5′-AGG ACC CAA GCA CCT TCT TT-3′ (forward) 5′-AGA CAG CAC GAG GCA TTT T-3′ (reverse)
IL-6	5′-TAG TCC TTC CTA CCC CAA CT-3′ (forward) 5′-TTG GTC CTT AGC CAC TCC TT-3′ (reverse)
TNF-*α*	5′-GTC TGT GCC TCA GCC TCT TC -3′ (forward) 5′-CCC ATT TGG GAA CTT CrC CT-3′ (reverse)
MCP-1	5′-GCA TCC ACG TGT TGG CTC A-3′ (forward) 5′-CTC CAG CCT ACT TCA TTG GGA TC-3′ (reverse)
MMP-7	5′-GTT TTT GAT GCT ATT GCT GA-3′ (forward) 5′-CCC ACA TTT GAC GTC CAG TCC AGA G-3′ (reverse)
MMP-9	5′-ACG ACA TAG ACG CCA TCC AGT-3′ (forward) 5′-AGG TAT AGT GGG ACG ACT GGG-3′ (reverse)
TGF-*β*	5′-TTG CTT CAG CTC CAC AGA GA-3′ (forward) 5′-TGG TTG TAG AGG GCA AGG AC-3′ (reverse)
GAPDH	5′-ACA GCA ACA GGG TGG TGG AC-3′ (forward) 5′-TTT GAG GGT GCA GCG AAC TT-3′ (reverse)

**Table 2:** Linear range, regression equation, *r*^2^, LODs, and LOQs for marker analytes (*n* = 3).

Analyte	Linear range (*μ*g/mL)	Regression equation^a^ (*y* = a*x* + *b*)	*r* ^2^	LOD^b^ (*μ*g/mL)	LOQ^c^ (*μ*g/mL)
Liquiritin apioside	0.47–30.00	*y* = 13919.31*x* + 1750.37	0.9999	0.09	0.28
Liquiritin	0.47–30.00	*y* = 23478.34*x* + 2764.20	1.0000	0.07	0.23
Narirutin	0.78–50.00	*y* = 16945.14*x* + 3555.47	1.0000	0.13	0.40
Hesperidin	0.78–50.00	*y* = 16439.25*x* + 2890.32	1.0000	0.09	0.26
Glycyrrhizin	0.47–30.00	*y* = 7344.40*x* + 533.61	1.0000	0.03	0.09
6-Gingerol	0.47–30.00	*y* = 6451.07*x* + 686.49	1.0000	0.03	0.09

^a^ *y*: peak area (mAU) of each analyte; *x*: concentration (*μ*g/mL) of each analyte. ^b^LOD = 3.3 × *σ*/*S*. ^c^LOQ = 10 × *σ*/*S* (where *σ* is the standard deviation of the *y*-intercept, and *S* is the slope of the calibration curve).

**Table 3:** Concentration of the six marker analytes in the Yijin-tang sample (*n* = 3).

Analyte	Batch no.					
	1		2		3	
	Mean (mg/g) ± SD^a^ (×10^−1^)	RSD (%)	Mean (mg/g) ± SD (×10^−1^)	RSD (%)	Mean (mg/g) ± SD (×10^−1^)	RSD (%)
Liquiritin apioside	6.16 ± 1.06	1.72	6.49 ± 0.80	1.23	6.29 ± 0.31	0.49
Liquiritin	3.22 ± 0.55	1.71	3.39 ± 0.35	1.03	3.28 ± 0.20	0.61
Narirutin	8.09 ± 1.15	1.42	8.51 ± 0.95	1.12	8.25 ± 0.71	0.86
Hesperidin	12.24 ± 1.98	1.62	12.88 ± 1.15	0.89	12.49 ± 1.02	0.81
Glycyrrhizin	6.77 ± 1.51	2.23	7.11 ± 0.43	0.61	6.88 ± 0.68	0.99
6-Gingerol	0.28 ± 0.0.04	1.48	0.28 ± 0.05	1.91	0.28 ± 0.02	0.71

^a^SD means standard deviation.

## Data Availability

The data generated from the findings of this study are included within the article.

## Abbreviations

COPD: Chronic obstructive pulmonary disease
COX-2: Cyclooxygenase-2
CS: Cigarette smoke
IL-1*β*: Interleukin-1
IL-6: Interleukin-6
iNOS: Inducible nitric oxide synthase
MCP-1: Monocyte chemoattractant protein-1
MMP-7: Matrix metallopeptidase-7
MMP-9: Matrix metallopeptidase-9
NC: Negative control
NF-*κ*B: Nuclear factor-kappa B
p.o.: Per os
RO: Roflumilast
TGF-*β*: Transforming growth factor-*β*
TNF-*α*: Tumor necrosis factor-*α*
s.c.: Subcutaneous
YJT: Yijin-tang.

## Supplementary Materials

Supplementary Materials Table S1 provides the composition of YJT. Table S2 provides chromatographic parameters of 6 marker components in YJT using HPLC. Table S3 provides the system suitability of the 6 marker components for the simultaneous analysis of YJT. Supplementary Figure 1 provides the chemical structures of reference standard compounds used for HPLC analysis of YJT.

## References

[1] Vestbo J., Hurd S. S., Rodriguez-Roisin R. (2012). The 2011 revision of the global strategy for the diagnosis, management and prevention of COPD (GOLD)-why and what?. *The Clinical Respiratory Journal*.

[2] Vestbo J., Edwards L. D., Scanlon P. D. (2011). Changes in forced expiratory volume in 1 second over time in COPD. *New England Journal of Medicine*.

[3] Curtis J. L., Freeman C. M., Hogg J. C. (2007). The immunopathogenesis of chronic obstructive pulmonary disease: insights from recent research. *Proceedings of the American Thoracic Society*.

[4] Kew K. M., Dias S., Cates C. J. (2014). Long-acting inhaled therapy (beta-agonists, anticholinergics and steroids) for COPD: a network meta-analysis. *Cochrane Database of Systematic Reviews*.

[5] Rabe K. F., Watz H. (2017). Chronic obstructive pulmonary disease. *The Lancet*.

[6] Alagha K., Palot A., Sofalvi T. (2014). Long-acting muscarinic receptor antagonists for the treatment of chronic airway diseases. *Therapeutic Advances in Chronic Disease*.

[7] Chen S., Hui Z. Z. Z. S. B. G. C. G. H. Z. D. W. Y., Hui Z. Z. Z. S. B. G. C. B. Z. C. B. W. Y. (2006). *Tai Ping Hui Min He Ji Ju Fang*.

[8] Heo J. (1994). *Donguibogam*.

[9] Yang H. J., Yim N.-H., Lee K. J. (2016). Simultaneous determination of nine bioactive compounds in Yijin-tang via high-performance liquid chromatography and liquid chromatography-electrospray ionization-mass spectrometry. *Integrative Medicine Research*.

[10] Zhang Q., Ye M. (2009). Chemical analysis of the Chinese herbal medicine Gan-Cao (licorice). *Journal of Chromatography A*.

[11] Kim K.-J., Choi J.-S., Kim K.-W., Jeong J.-W. (2013). The anti-angiogenic activities of glycyrrhizic acid in tumor progression. *Phytotherapy Research*.

[12] Hirata T., Fujii M., Akita K. (2009). Identification and physiological evaluation of the components from citrus fruits as potential drugs for anti-corpulence and anticancer. *Bioorganic & Medicinal Chemistry*.

[13] Nagai T., Kiyohara H., Munakata K. (2002). Pinellic acid from the tuber of Pinellia ternata Breitenbach as an effective oral adjuvant for nasal influenza vaccine. *International Immunopharmacology*.

[14] Dugasani S., Pichika M. R., Nadarajah V. D., Balijepalli M. K., Tandra S., Korlakunta J. N. (2010). Comparative antioxidant and anti-inflammatory effects of [6]-gingerol, [8]-gingerol, [10]-gingerol and [6]-shogaol. *Journal of Ethnopharmacology*.

[15] Cheng S., Eliaz I., Lin J., Sliva D., Sliva D. (2013). Triterpenes from Poria cocos suppress growth and invasiveness of pancreatic cancer cells through the downregulation of MMP-7. *International Journal of Oncology*.

[16] Lee Y.-H., Lee N.-H., Bhattarai G. (2013). Anti-inflammatory effect of pachymic acid promotes odontoblastic differentiation via HO-1 in dental pulp cells. *Oral Diseases*.

[17] Kh L. (2012). *The Dispensatory on the Visual and Organoleptic Examination of Herbal Medicine*.

[18] Kim S.-S., Kim J.-H., Shin H.-K., Seo C.-S. (2013). Simultaneous analysis of six compounds in Yijin-tang by HPLC-PDA. *Herbal Formula Science*.

[19] Vicencio A. G., Lee C. G., Cho S. J. (2004). Conditional overexpression of bioactive transforming growth factor-*β*1 in neonatal mouse lung. *American Journal of Respiratory Cell and Molecular Biology*.

[20] Park H.-S., Wijerathne C. U. B., Jeong H.-Y., Seo C.-S., Ha H., Kwun H.-J. (2018). Gastroprotective effects of Hwanglyeonhaedok-tang against Helicobacter pylori-induced gastric cell injury. *Journal of Ethnopharmacology*.

[21] Zhang Y., Zhou S., Zhou J., Wang D., Zhou T. (2019). Regulation of NF-*κ*B/MAPK signaling pathway attenuates the acute lung inflammation in Klebsiella pneumonia rats by mollugin treatment. *Microbial Pathogenesis*.

[22] Schuliga M. (2015). NF-kappaB signaling in chronic inflammatory airway disease. *Biomolecules*.

[23] Grzela K., Litwiniuk M., Zagorska W., Grzela T. (2016). Airway remodeling in chronic obstructive pulmonary disease and asthma: the role of matrix metalloproteinase-9. *Archivum Immunologiae et Therapiae Experimentalis*.

[24] Stratton K., Shetty P., Wallace R., Bondurant S., Stratton K., Shetty P., Wallace R., Bondurant S. (2001). *Clearing the Smoke: Assessing the Science Base for Tobacco Harm Reduction*.

[25] Yuan F., Fu X., Shi H., Chen G., Dong P., Zhang W. (2014). Induction of murine macrophage M2 polarization by cigarette smoke extract via the JAK2/STAT3 pathway. *PLoS One*.

[26] Cipollina C., Di Vincenzo S., Gerbino S., Siena L., Gjomarkaj M., Pace E. (2014). Dual anti-oxidant and anti-inflammatory actions of the electrophilic cyclooxygenase-2-derived 17-oxo-DHA in lipopolysaccharide- and cigarette smoke-induced inflammation. *Biochimica et Biophysica Acta (BBA)-General Subjects*.

[27] Blake D. J., Singh A., Kombairaju P. (2010). Deletion ofKeap1in the lung attenuates acute cigarette smoke-induced oxidative stress and inflammation. *American Journal of Respiratory Cell and Molecular Biology*.

[28] Wang Z., Zheng T., Zhu Z. (2000). Interferon *γ* induction of pulmonary emphysema in the adult murine lung. *Journal of Experimental Medicine*.

[29] Malaviya R., Laskin J. D., Laskin D. L. (2017). Anti-TNF*α* therapy in inflammatory lung diseases. *Pharmacology & Therapeutics*.

[30] Broekhuizen R., Wouters E. F., Creutzberg E. C., Schols A. M. (2006). Raised CRP levels mark metabolic and functional impairment in advanced COPD. *Thorax*.

[31] Lappalainen U., Whitsett J. A., Wert S. E., Tichelaar J. W., Bry K. (2005). Interleukin-1*β* causes pulmonary inflammation, emphysema, and airway remodeling in the adult murine lung. *American Journal of Respiratory Cell and Molecular Biology*.

[32] Di Stefano A., Coccini T., Roda E. (2018). Blood MCP-1 levels are increased in chronic obstructive pulmonary disease patients with prevalent emphysema. *International Journal of Chronic Obstructive Pulmonary Disease*.

[33] Hamid Q., Cosio M., Lim S. (2004). Inflammation and remodeling in chronic obstructive pulmonary disease. *The Journal of Allergy and Clinical Immunology*.

[34] Jeffery P. K. (2004). Remodeling and inflammation of bronchi in asthma and chronic obstructive pulmonary disease. *Proceedings of the American Thoracic Society*.

[35] Elkington P. T. G., Friedland J. S. (2006). Matrix metalloproteinases in destructive pulmonary pathology. *Thorax*.

[36] Shapiro S. D. (1999). The macrophage in chronic obstructive pulmonary disease. *American Journal of Respiratory and Critical Care Medicine*.

[37] Kang M. J., Oh Y. M., Lee J. C. (2003). Lung matrix metalloproteinase-9 correlates with cigarette smoking and obstruction of airflow. *Journal of Korean Medical Science*.

[38] Brajer B., Batura-Gabryel H., Nowicka A., Kuznar-Kaminska B., Szczepanik A. (2008). Concentration of matrix metalloproteinase-9 in serum of patients with chronic obstructive pulmonary disease and a degree of airway obstruction and disease progression. *Journal of Physiology & Pharmacology*.

[39] Rosas I. O., Richards T. J., Konishi K. (2008). MMP1 and MMP7 as potential peripheral blood biomarkers in idiopathic pulmonary fibrosis. *PLoS Medicine*.

[40] Montaño M., Sansores R. H., Becerril C. (2014). FEV1 inversely correlates with metalloproteinases 1, 7, 9 and CRP in COPD by biomass smoke exposure. *Respiratory Research*.

[41] Shin I.-S., Park J.-W., Shin N.-R. (2014). Melatonin reduces airway inflammation in ovalbumin-induced asthma. *Immunobiology*.

[42] Perng D. W., Chang K. T., Su K. C. (2011). Matrix metalloprotease-9 induces transforming growth factor-beta (1) production in airway epithelium via activation of epidermal growth factor receptors. *Life Sciences*.

[43] Di Stefano A., Sangiorgi C., Gnemmi I. (2018). TGF-*β* signaling pathways in different compartments of the lower airways of patients with stable COPD. *Chest*.

[44] Leask A., Abraham D. J. (2004). TGF‐*β* signaling and the fibrotic response. *The FASEB Journal*.

[45] Shin N.-R., Ko J.-W., Park S.-H. (2017). Protective effect of HwangRyunHaeDok-Tang water extract against chronic obstructive pulmonary disease induced by cigarette smoke and lipopolysaccharide in a mouse model. *Journal of Ethnopharmacology*.

[46] Lee J., Taneja V., Vassallo R. (2012). Cigarette smoking and inflammation. *Journal of Dental Research*.

[47] Wang W., Li X., Xu J. (2015). Exposure to cigarette smoke downregulates *β*2-adrenergic receptor expression and upregulates inflammation in alveolar macrophages. *Inhalation Toxicology*.

