# Supplementary material for: Yijin-Tang Attenuates Cigarette Smoke and Lipopolysaccharide-Induced Chronic Obstructive Pulmonary Disease in Mice
Source: Evid Based Complement Alternat Med. 2022 Jan 5;2022:7902920. doi: 10.1155/2022/7902920 (PMC8754600; doi:10.1155/2022/7902920)
Supplement: Supplementary Materials — Table S1 provides the composition of YJT. Table S2 provides chromatographic parameters of 6 marker components in YJT using HPLC. Table S3 provides the system suitability of the 6 marker components for the simultaneous analysis of YJT. Supplementary Figure 1 provides the chemical structures of reference standard compounds used for HPLC analysis of YJT. [file 7902920.f1.zip › 7902920.f1/supplementary table.docx]

**Table S1** Composition of Yijin-tang

| **Herbal medicine** | **Scientific name** | **Family** | **Using parts** | **Origin** | **Amount (g)** | **Ratio (%)** |
| --- | --- | --- | --- | --- | --- | --- |
| Pinelliae Tuber | *Pinellia ternate* Breitenbach | Araceae | Tuber | China | 1272.74 | 36.37 |
| Citri Unshius Pericarpium | *Citrus unshiu* Markovich | Rutaceae | Pericarp | Jeju, Korea | 636.36 | 18.18 |
| Poria Sclerotium | *Poria cocos* Wolf | Polyporaceae | Sclerotium | Bonghwa, Korea | 636.36 | 18.18 |
| Glycyrrhizae Radix et Rhizoma | *Glycyrrhiza uralensis* Fischer | Leguminosae | Root and rhizome | China | 318.18 | 9.09 |
| Zingiberis Rhizoma Recens | *Zingiber officinale* Roscoe | Zingiberaceae | Rhizome | Seosan, Korea | 636.36 | 18.18 |
|  |  |  |  | Sum | 3500.00 | 100.00 |

**Table S2** Chromatographic parameters for quantitative analysis of the 6 marker components in Yijin-tang sample using HPLC

| Chromatographic parameter | |
| --- | --- |
| Column | SunFire C_18_ analytical column (250 × 4.6 mm, 5 μm) |
| Detector | PDA (220, 255, 275, 280 and 290 nm) |
| Flow rate (mL/min) | 1.0 |
| Injection volume (μL) | 10.0 |
| Column temperature (°C) | 40.0 |
| Mobile phase | A: 0.1% (v/v) aqueous formic acid  B: 0.1% (v/v) formic acid in acetonitrile |
| Gradient elution | \| Time (min) \| A (%) \| B (%) \| \| --- \| --- \| --- \| \| 0 \| 95 \| 5 \| \| 30 \| 40 \| 60 \| \| 40 \| 0 \| 100 \| \| 45 \| 0 \| 100 \| \| 50 \| 95 \| 5 \| \| 60 \| 95 \| 5 \| |

**Table S3** System suitability of the 6 marker components for the simultaneous analysis of Yijin-tang sample using HPLC

| Compound | *k*′ | *α* | *N* | *Rs* | *Tf* |
| --- | --- | --- | --- | --- | --- |
| Liquiritin apioside | 4.65 | 1.03 | 36077.78 | 2.64 | 1.08 |
| Liquiritin | 4.79 | 1.03 | 49821.84 | 2.64 | 1.08 |
| Naritutin | 5.07 | 1.06 | 54415.31 | 5.96 | 1.18 |
| Hesperidin | 5.38 | 1.06 | 64833.89 | 6.69 | 1.12 |
| Glycyrrhizin | 9.56 | 1.07 | 105447.80 | 9.98 | 1.08 |
| 6-Gingerol | 10.20 | 1.07 | 214628.89 | 9.98 | 1.03 |
