# Supplementary figures and images for: Yijin-Tang Attenuates Cigarette Smoke and Lipopolysaccharide-Induced Chronic Obstructive Pulmonary Disease in Mice
Source: Evid Based Complement Alternat Med. 2022 Jan 5;2022:7902920. doi: 10.1155/2022/7902920 (PMC8754600; doi:10.1155/2022/7902920)

**Supplementary fig. 1**

**
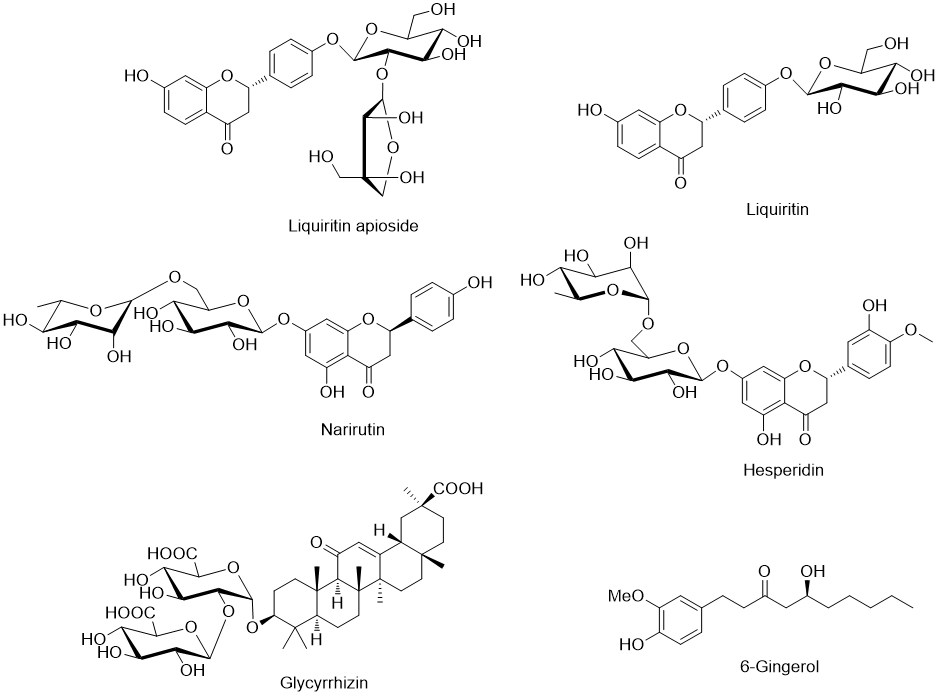
**

Supplement: Supplementary Materials — Table S1 provides the composition of YJT. Table S2 provides chromatographic parameters of 6 marker components in YJT using HPLC. Table S3 provides the system suitability of the 6 marker components for the simultaneous analysis of YJT. Supplementary Figure 1 provides the chemical structures of reference standard compounds used for HPLC analysis of YJT. [file 7902920.f1.zip › 7902920.f1/supplementary figure.docx]
